# Spatial distribution characteristics and evaluation of soil pollution in coal mine areas in Loess Plateau of northern Shaanxi

**DOI:** 10.1038/s41598-022-20865-6

**Published:** 2022-09-30

**Authors:** Na Wang, Yuhu Luo, Zhe Liu, Yingying Sun

**Affiliations:** 1 Institute of Land Engineering and Technology, Shaanxi Provincial Land Engineering Construction Group Co., Ltd., Xi’an, 710075 China; 2 Shaanxi Provincial Land Engineering Construction Group Co., Ltd., Xi’an, 710075 China; 3 Key Laboratory of Degraded and Unused Land Consolidation Engineering, the Ministry of Natural Resources, Xi’an, 710075 China; 4 Shaanxi Provincial Land Consolidation Engineering Technology Research Center, Xi’an, 710075 China

**Keywords:** Ecology, Environmental sciences, Environmental social sciences, Natural hazards

## Abstract

The ecological environment in Loess Plateau of Northern Shaanxi is fragile, so the soil pollution caused by the exploitation of coal resources cannot be ignored. With Shigetai Coal Mine in Loess Plateau of Northern Shaanxi as the object of study for field survey and sampling, the content of heavy metals in soil is analyzed, the environmental pollution in the research area is evaluated by the single factor pollution index method, comprehensive pollution index method and potential ecological risk index method, and the spatial distribution characteristics of heavy metals are discussed by the geostatistics method. According to the study results, the average contents of heavy metals Hg, Cd, Pb and Cr are 2.03, 1.36, 1.11 and 1.23 times of the soil background values in Shaanxi Province respectively and the average contents of other heavy metals are lower than the soil background values in Shaanxi Province; Hg and Cd show moderate variation while As, Pb, Cr, Zn, Ni and Cu show strong variation; the skewness coefficients and kurtosis coefficient of Cd, As and Cu in the soil within the research area are relatively high, and these elements are accumulated in large amounts. Single factor pollution index (Pi) and potential ecological risk index (E) indicate that heavy metal Hg is the main pollution factor and mainly distributed in the east and north of the research area. The comprehensive index of potential ecological risk (RI) of the research area is 1336.49, showing an extremely high ecological risk, and the distribution characteristics of potential ecological risk are consistent with that of potential ecological risk index (E) of Hg. The results of ecological risk warning show that Hg is in a slight warning status, while Cd, Pb and Cr are in a warning status. The areas with high ecological risk warning values are mainly distributed in the east and north, and the whole research area shows relatively obvious zonal distribution law. The soil is disturbed greatly during the coal mining, so the ecological governance of the mine area shall adapt to the local natural conditions and regional environmental characteristics and follow the principle of “adjusting governance measures based on specific local conditions and classifications”. An environmentally sustainable governance manner shall be adopted to realize the protection of the ecological environment and high-quality development of coal resources.

## Introduction

In recent years, the cultivated land area has been reduced and the natural environment has been seriously polluted due to the industrial revolution, rapid population growth, rural–urban migration and unplanned and uncontrolled urbanization^1^. With the high toxicity, long-term retention, persistent bioavailability and recalcitrance, pollutants such as heavy metals and metalloids (mercury, lead, zinc, copper, cadmium, chromium, nickel, arsenic) are considered to be the main harmful trace elements^2–4^. The sources of heavy metals in soil mainly include natural factors and human factors. The natural factor mainly refers to the rock weathering in the process of soil formation, and many studies showed that the concentration of the heavy metals formed by the natural factor is usually harmless to the ecological environment^5^. Human activities, such as exploitation of mineral resources, metal processing and smelting, chemical production, factory discharge and sewage irrigation, have been proved to be the main sources of heavy metal pollution^6–8^. According to statistics, the land area polluted by heavy metals in China is more than 10 million hectares, seriously threatening the agricultural development and quality and safety of ecological environment in China^9,10^. In addition, excessive heavy metals may enter the human body through the food chain, causing a variety of human diseases^11^. For example, excessive lead in the human body will damage their nervous and immune systems, and long-term exposure to cadmium will lead to potential risks of lung cancer and fracture, long-term intake of high concentrations of copper and zinc will damage human pancreas, liver and kidney and lower the HDL cholesterol level^12^, long-term consumption of crops and vegetables grown on the soil polluted by the arsenic will increase the risks of skin cancer, bladder cancer and lung cancer^13,14^. Therefore, in order to protect the ecosystem and human health, it is essential to evaluate the distribution, sources and potential environmental risks of heavy metals in soil.

Mining is one of the most polluted activities in the world. It is well-known that mining activities will lead to the loss of surrounding biodiversity^15,16^. In particular, mining activities will cause a wide range of landform disturbances, such as the destruction of geological continuity on the surface, soil pollution, hydrological impact on runoff capacity, and changes in landscape morphology. Excavation and overburden deposition aggravate the change of surface topography and cause the degeneration of the aquifers^17^. After the founding of new China, in order to vigorously develop the economy, China adopted an extensive economic development model, that is, treatment after pollution, which led to serious environmental problems. The open-pit coal mining is one of these models. No effective preventive and protective measures have been taken in the process of coal mining, resulting in serious environmental problems around the mine area. Heavy metal is one of the main pollutants in the coal mine area. According to statistics, about 3.2 million hectares of land in China were affected by mining activities prior to 1996, including cultivated land, woodland and pasture^18^. Many previous studies have shown that heavy metals are released into farmland soil, surface water and plant leaves around the mine area during coal mining and processing, endangering human health through the food chain^19,20^. In the process of coal mining, such as ore concentration, mineral extraction, topsoil stripping, tailings accumulation, wastewater treatment and transportation, a large amount of heavy metal dust and dusty wastewater are generated around the mine area^21^. After being mixed with soil, these refractory heavy metals migrate to the water environment through surface runoff, which amplifies their toxicity in the ecological environment^22^. In addition, long-term overexploitation causes land subsidence, soil erosion and deterioration of groundwater quality, seriously disturbing the local natural environment^23^. It is well-known that coal gangue and fly ash contain a variety of toxic heavy metal elements, which are released into the environment through coal transportation, smoke and dust discharging from coal-fired power plants, sewage discharging from mine areas and other coal-related industry activities. In addition, the long-term accumulation of coal gangue and fly ash is another way to release toxic elements into the soil^24^. *Masto *et al.^25^ and other^26^ researchers showed that the coal dust contains heavy metals such as iron, zinc, manganese, copper, lead, chromium, nickel, strontium, zirconium and arsenic, which are seeped into soil and even groundwater through surface runoff. According to statistics, the average concentrations of arsenic in bituminous coal and lignite in the world are 9.0 and 7.4 mg/kg, respectively^27^. Chen et al.^28^ found that the contents of arsenic, selenium, mercury and antimony discharged into the atmosphere from coal-fired power plants are 236, 637, 172 and 33 tons, respectively.

With rich coal, natural gas and oil resources, Northern Shaanxi is an important energy and chemical base in Shaanxi Province. Its ecosystem is very fragile and sensitive to environmental impact^29^. The geological tectonic unit of the mine area belongs to Ordos platform slope of North China platform and the north-central part of northern Shaanxi platform depression. No magmatic rock formation, magmatism and volcanic eruption occur and earthquakes are rare in this area. It borders the sandy grassland on the southern margin of Maowusu Desert in its north, and borders the hinterland of Loess Plateau in its south, with crisscrossed gullies and crisscross hills and ridges. At present, the study on heavy metals in mining industry mainly focuses on the evaluation, nature, mechanism, ecological improvement and biological effects of heavy metal pollution^30^. There are only sporadic research reports on Shenmu Mine Area, a super-large energy base located in water-wind erosion area and few reports on the spatial distribution and potential pollution evaluation of heavy metals in soil in this area. Geostatistics is used to predict the extent of soil and groundwater pollution as well as to calculate the risk in active or abandoned mining, waste disposal and urban sites, by accounting for the spatial distribution and uncertainty of the estimates. It facilitates quantification of the spatial characteristics of soil parameters and enables spatial interpolation^31–33^.

Therefore, with Shigetai Coal Mine in Shenmu as the research area, the pollution level of toxic substances is evaluated and their spatial distribution characteristics are discussed by using the single factor pollution index method, Nemerow pollution index method and potential ecological evaluation method based on GIS technology and geostatistical theory, in order to provide scientific support and basis for environmental management and standardization.

## Materials and methods

### Overview of the research area

**Figure 1:**
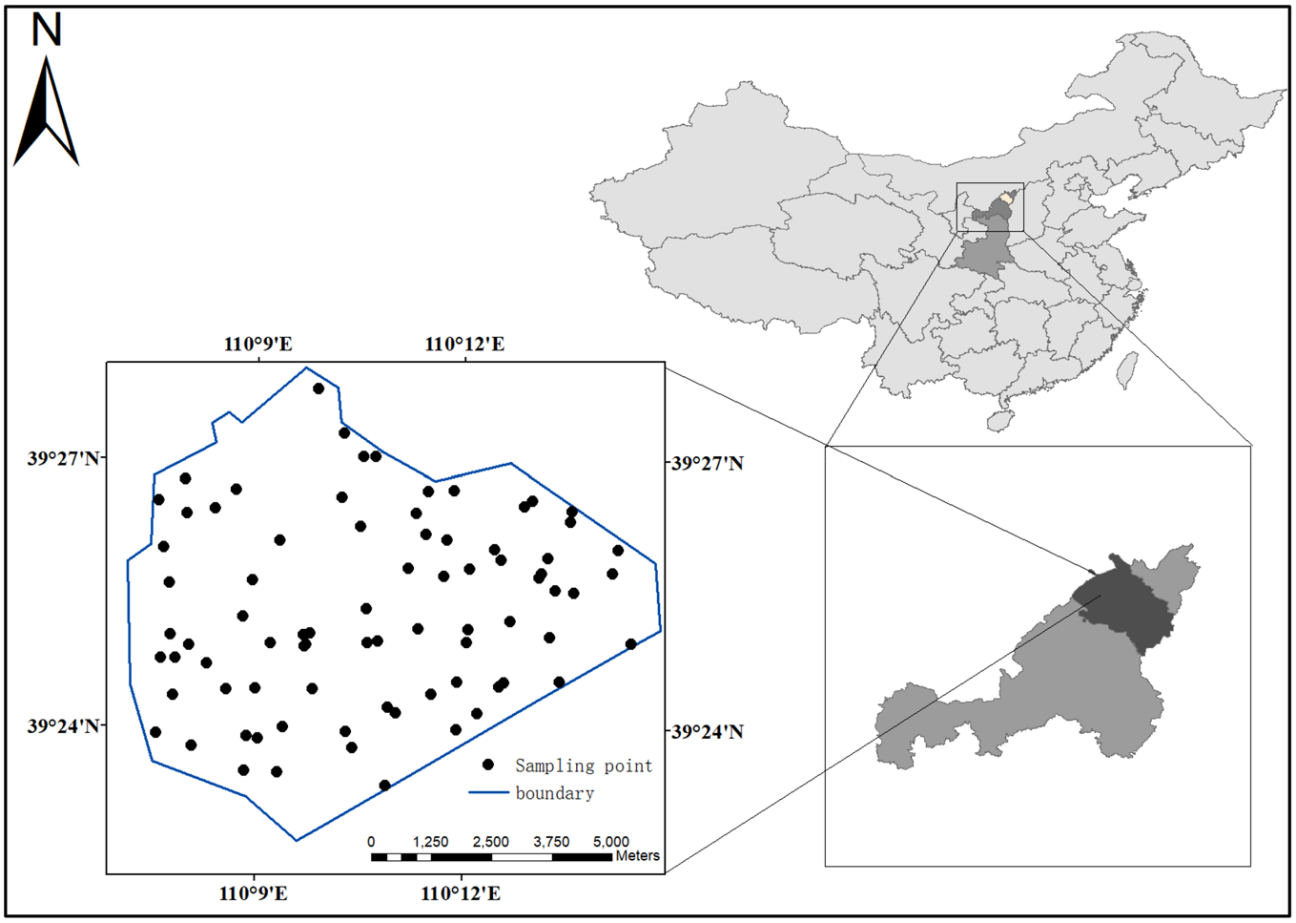
Location of the research area and the distribution of sampling points (created by Arcgis 10.8, http://desktop.arcgis.com/cn/).

The research area is located in Shigetai Coal Mine in the north of Loess Plateau of Northern Shaanxi on the southern margin of Maowusu Desert (Fig. 1). The coal field is bordered by Ulanmulun River in the west, Halagou coal field in the south, Battuta coal field in the north, Qigaigou and Shaanxi-Inner Mongolia border in the east. The geographical coordinates of the area are: 110° 09′ 41″ E–110° 18′ 35″ E and 39° 17′ 02″ N–39° 35′ 16″ N. The research area is dominated by a semi-arid continental climate in the mid-temperate zone, with cold winter and hot summer. The temperature difference between day and night is large, with the maximum value of about 20℃. The maximum frozen soil depth is 146 cm and the maximum snow depth is 12 cm from November to March of the following year. The monsoon period lasts from the beginning of January to the beginning of May and the prevailing wind direction is northwest wind. The annual average wind speed is 2.5 m/s and the maximum wind speed is 25 m/s. The annual average temperature is 8.5℃, the extreme maximum temperature is 38.9 ℃, the extreme minimum temperature is 28.5℃, and the annual average precipitation is 434.10 mm, which mainly occurs from July to September. The average annual evaporation is 1907.2–2122.7 mm, which is about 4–5 times of the precipitation.

Shigetai Coal Mine, one of the main production mines of Shendong Coal Group, was officially put into operation on January 15, 2006, with a geological reserve of 893 million tons and a recoverable reserve of 657 million tons. Characterized by low ash content, low sulfur content, low phosphorus content, medium to high calorific value in quality, the coal mined belongs to long flame coal with high volatile component and non-caking coal, which is the high-quality steam coal, chemical and metallurgical coal.

### Sample collection

The sampling time is June 2020 and the soil sampling depth is 0–30 cm. One sample of about 0.5 kg is collected at the sampling point first, and then the 0.5 kg soil sample is collected at the other two sampling points within 2 m near this point respectively. Mix three soil samples evenly, take 500 g with quartering method to form one sample, and put it into a cloth bag. Put the collected samples into a cloth bag first to drain the most of the water before putting them into a polyethylene bag. Dry the sample in the air, and take some samples dried to be screened by 0.149 mm sieve, and then test Hg, Cd, As, Pb, Cr, Zn, Ni and Cu. See Fig. 1 for the location of the research area and the distribution of sampling points.

### Test method

See Table 1 for test indexes of heavy metals. The parallel test is carried out on each sample for three times. During the test, the quality shall be controlled against the national standard soil reference substance (GSS-12) and duplicate samples. The test results show that the recovery rates of all elements are within the allowable range.

**Table 1:** Executive standard for test of heavy metal contents.

Element	Test basis	Test limit (mg/kg)	Test Equipment
Hg	GB/T 22105.1-2008	0.002	BAF-2000 atomic fluorescence spectrophotometer
Cd	GB/T 17141-1997	0.01	SOLAAR M6 atomic absorption spectrometer
As	GB/T 22105.2-2008	0.01	BAF-2000 atomic fluorescence spectrophotometer
Pb	GB/T 17141-1997	0.10	SOLAAR M6 atomic absorption spectrometer
Cr	HJ 350-2007	0.40	ICP-5000 inductively coupled plasma emission spectrometer
Zn	HJ 350-2007	0.10	ICP-5000 inductively coupled plasma emission spectrometer
Ni	HJ491-2019	3.0	SOLAAR M6 atomic absorption spectrometer
Cu	HJ491-2019	1.0	SOLAAR M6 atomic absorption spectrometer

### Evaluation methods

#### Nemerow pollution index method

With the soil background value in Shaanxi Province as a reference, the soil pollution status is evaluated by using the single factor pollution index method ($P_{i}$) and Nemerow pollution index method (NPI). The calculation equation is:

1 $$P_{i} = \frac{{C_{i} }}{{C_{n}^{i} }}$$

2 $$NPI = \sqrt {\frac{{P_{iave}^{2} + P_{i\max }^{2} }}{2}}$$

where, $C_{i}$ is the measured content of heavy metal i (mg/kg); $C_{n}^{i}$ is the background value of heavy metal i (mg/kg); $P_{i}$ is the single factor pollution index of heavy metal i; $NPI$ is Nemerow pollution index, $P_{iave}$ is the average value of each single factor pollution index of heavy metals, and $P_{i\max }$ is the maximum value of single factor pollution index of heavy metals. See Table 2 for pollution index classification standard.

**Table 2:** Classification standard for single factor pollution index and Nemerow pollution index.

Pollution grade	$P_{i}$	Pollution level	$NPI$	Pollution level
1	$P_{i}$ ≤ 1	Without pollution	$NPI$ ≤ 0.7	Clean/safe
2	1 < $P_{i}$ ≤ 2	Mild pollution	0.7 < $NPI$ ≤ 1	Almost clean/close to the warning line
3	2 < $P_{i}$ ≤ 3	Moderate pollution	1 < $NPI$ ≤ 2	Mild pollution
4	$P_{i}$ > 3	Heavy pollution	2 < $NPI$ ≤ 3	Moderate pollution
5	/	/	$NPI$ > 3	Heavy pollution

#### Potential ecological risk index method

Potential ecological risk index method can reflect the pollution level of a single heavy metal element and the comprehensive effect of all heavy metal elements from the perspective of the biological toxicity of heavy metals. The calculation equation is:

3 $$RI_{j} = \sum\limits_{i = 1}^{n} {E_{j}^{i} } = \sum\limits_{i = 1}^{n} {T_{i} \times C_{j}^{i} } = \sum\limits_{i = 1}^{n} {T_{i} \times \frac{{c_{j}^{i} }}{{c_{r}^{i} }}}$$

where: $RI_{j}$ is the comprehensive potential ecological risk index of multiple heavy metals from the sampling point j, $E_{j}^{i}$ is the single potential ecological risk index of heavy metal i from the sampling point j, $C_{j}^{i}$ is the pollution index of heavy metal i from the sampling point j, $c_{j}^{i}$ is the measured concentration of heavy metal i from the sampling point j, $c_{r}^{i}$ is the reference value of heavy metal i (the background value of soil environment in Shaanxi is taken as the reference value in this study), and $T_{i}$ is the toxicity coefficient of heavy metal i^34,35^. See Table 3 for $c_{r}^{i}$ and $T_{i}$.

**Table 3:** Environmental background values and toxic-response parameters of heavy metals in the soil.

Element	Hg	Cd	As	Pb	Cr	Zn	Ni	Cu
$c_{r}^{i}$	0.063	0.76	11.1	21.4	62.5	69.4	28.8	21.4
$T_{i}$	40	30	10	5	2	1	5	5

#### Ecological risk warning index method

In this research, the warning for the ecological risks of heavy metal pollution in open-pit coal mining areas is evaluated based on the ecological risk warning index ($I_{ER}$) proposed by Rapant and Kordik^36^. The calculation equation is:

4 $$I_{ER} = \sum\limits_{i = 1}^{n} {I_{ERj} } = \sum\limits_{i = 1}^{n} {(P_{i} - 1)}$$

where: $I_{ER}$ is the ecological risk warning index, $I_{{ER{\text{j}}}}$ is the ecological risk index of the i^th^ heavy metal, and $Pi$ is the pollution index of the heavy metal i. See Table 4 for classification of pollution risk levels of $RI$ and $I_{ER}$.

**Table 4:** Classification of risk degree of RI and I_ER_.

Class	$RI$		$I_{ER}$	
I	$E$ ≤ 40; $RI$ ≤ 150	Low	$I_{ER}$ ≤ 0	No
II	40 < $E$ ≤ 80; 150 < $RI$ ≤ 300	Medium	0 < $I_{ER}$ ≤ 1	Early
III	80 < $E$ ≤ 160; 300 < $RI$ ≤ 600	High	1 < $I_{ER}$ ≤ 3	Low
IV	160 < $E$ ≤ 320; 600 < $RI$ ≤ 1200	Very high	3 < $I_{ER}$ ≤ 5	Medium
V	$E$ > 320; $RI$ > 1200	Extremely high	$I_{ER}$ > 5	High

### Geostatistics method

Geostatistics is an effective method to study the spatial distribution structure characteristics of regional variables. Its basic tool is a semivariate function that can be estimated by the following formula^37^:

5 $$\gamma (h) = \frac{1}{2N(h)}\sum\limits_{i = 1}^{N(h)} {[Z(x_{i} ) - Z(x_{i} + h)]^{2} }$$

where, γ(h) is the variation function, Z(x) is the value of the regionalised variable at the sampling point x, N(h) is the number of pairs with interval h and h is the interval, which is called the lag distance. Variograms can reflect and describe many properties of regionalised variables, and it is an important tool to analyze their spatial variation.

### Data analysis

The data is summarized and processed by SPSS10.0 software, and the spatial distribution of single factor pollution index (Pi), Nemerow pollution index (NPI), potential ecological risk index (E), RI and ecological risk warning index (IER) is determined by ArcGIS 10.8 Kriging interpolation method.

## Results and discussion

### Analysis of contents of heavy metals in wasteland soil

The test results show (Table 5) that the contents of Hg, Cd, As, Pb, Cr, Zn, Ni and Cu in the surface soil within Shigetai Coal Mine vary from 0.043 to 0.255, 0.44 to 2.23, 2.66 to 18.40, 11.80 to 42.80, 40.50 to 118.60, 18.90 to 70.10, 4.31 to 28.10, 4.96 to 46.25 mg/kg, respectively; the average contents of Hg, Cd, As, Pb, Cr, Zn, Ni and Cu are 0.128, 1.03, 4.73, 23.08, 76.22, 46.94, 16.11 and 12.10 mg/kg, respectively. The average contents of Hg, Cd, Pb and Cr in soil within the research area are 2.03, 1.36, 1.11 and 1.23 times of the soil background values in Shaanxi Province, respectively. The average contents of As, Zn and Cu are lower than the soil background value in Shaanxi Province, but the maximum contents of these three elements are 1.65, 1.01 and 2.16 times of the soil background values in Shaanxi Province, respectively. It is reported that the average concentration of lead in agricultural soil affected by coal mines is relatively high (433 mg kg^−1^)^38^. Lead is usually related to minerals in coal and occurs mainly in the form of sulfide such as PbS and PbSe^39^. In addition, aluminosilicate and carbonate also contain lead^40^. Chromium is a non-volatile element, which is related to aluminosilicate minerals^41^. In the mining process, chromium may be accumulated in coal, gangue or other tailings, and then enter the soil or water body through rain leaching^42^.

**Table 5:** Statistics of contents of heavy metals in wasteland soil (n = 79).

Parameter	Hg	Cd	As	Pb	Cr	Zn	Ni	Cu
Minimum (mg/kg)	0.043	0.44	2.66	11.80	40.50	18.90	4.31	4.96
Maximum (mg/kg)	0.255	2.23	18.40	42.80	118.60	70.10	28.10	46.25
Average (mg/kg)	0.128	1.03	4.73	23.80	76.22	46.94	16.11	12.10
Coefficient of variation (CV)	0.050	0.37	2.81	7.46	18.00	13.51	5.44	5.64
Skewness coefficient	0.389	0.36	0.60	0.31	0.24	0.29	0.34	0.47
Kurtosis coefficient	0.808	1.91	1.39	0.76	0.36	− 0.36	− 0.01	2.90
Background value of Shaanxi soil (mg/kg)	0.063	0.76	11.1	21.4	62.5	69.4	28.8	21.4

The coefficient of variation (CV) of Hg and Cd contents in soil within the research area is 0.050 and 0.37, respectively, with moderate variation, indicating that the content of these two heavy metals is less affected by the external factors; the coefficient of variation (CV) of As, Pb, Cr, Zn, Ni and Cu contents is 2.81, 7.46, 18.00, 13.51, 5.44 and 5.64, respectively, with strong variation (CV > 0.50)^43^, indicating that the content of these eight heavy metals may be affected by some local pollution sources. The skewness coefficient (SK) ranges from − 3 to 3, and the larger its absolute value, the greater its skewness. When SK > 0, it is positive skewness; when SK < 0, it is negative skewness. Kurtosis coefficient is the characteristic value representing the peak value of probability density distribution curve at the average value^44^. The skewness coefficient and kurtosis coefficient of Cd, As and Cu elements in the soil within the research area are relatively high, indicating that these three elements are accumulated in the soil within the research area in large amounts.

### Characteristics of heavy metal pollution in the research area

It can be seen from Table 6 that the average single factor pollution index (P_i_) of heavy metals in the surface soil within the research area is Hg(2.03), Cr(1.22), Cd(1.14), Pb(1.11), Zn(0.68), As(0.59), Cu(0.57) and Ni(0.56) in descending order.

**Table 6:** Ecological risk of heavy metals in the surface soil within the research area.

Parameter	P_i_								NPI
	Hg	Cd	As	Pb	Cr	Zn	Ni	Cu	
Minimum	0.68	0.57	0.24	0.55	0.65	0.27	0.15	0.23	0.80
Maximum	4.05	2.94	1.66	2.00	1.92	1.01	0.98	2.16	3.20
Average	2.03	1.14	0.59	1.11	1.22	0.68	0.56	0.57	1.64
Pollution class	III	II	I	II	II	I	I	I	III

The average value of each element in the research area is at the mild pollution level or above, Hg is at a moderate pollution level, Cd, Pb and Cr are at the mild pollution level, and other heavy metals are at the clean level. Among them, the sampling points with the single pollution index of element Hg at moderate pollution level account for 21.52% of the total number of sampling points, and the sampling points with the single pollution index of Cd, Pb and Cr elements at the mild pollution level account for 43.04%, 55.70% and 77.22% of the total number of sampling points, respectively. NPI of eight heavy metal elements in the surface soil within the research area is between 0.80 and 3.20, with an average value of 1.64, so it is at the mild pollution level. The results show that the heavy metal elements in the surface soil within the research area are affected by human activities, and Hg is the most important pollution factor. This is consistent with the research result obtained by *Li *et al.^45^.

### Spatial distribution pattern of heavy metal pollution in soil

**Figure 2:**
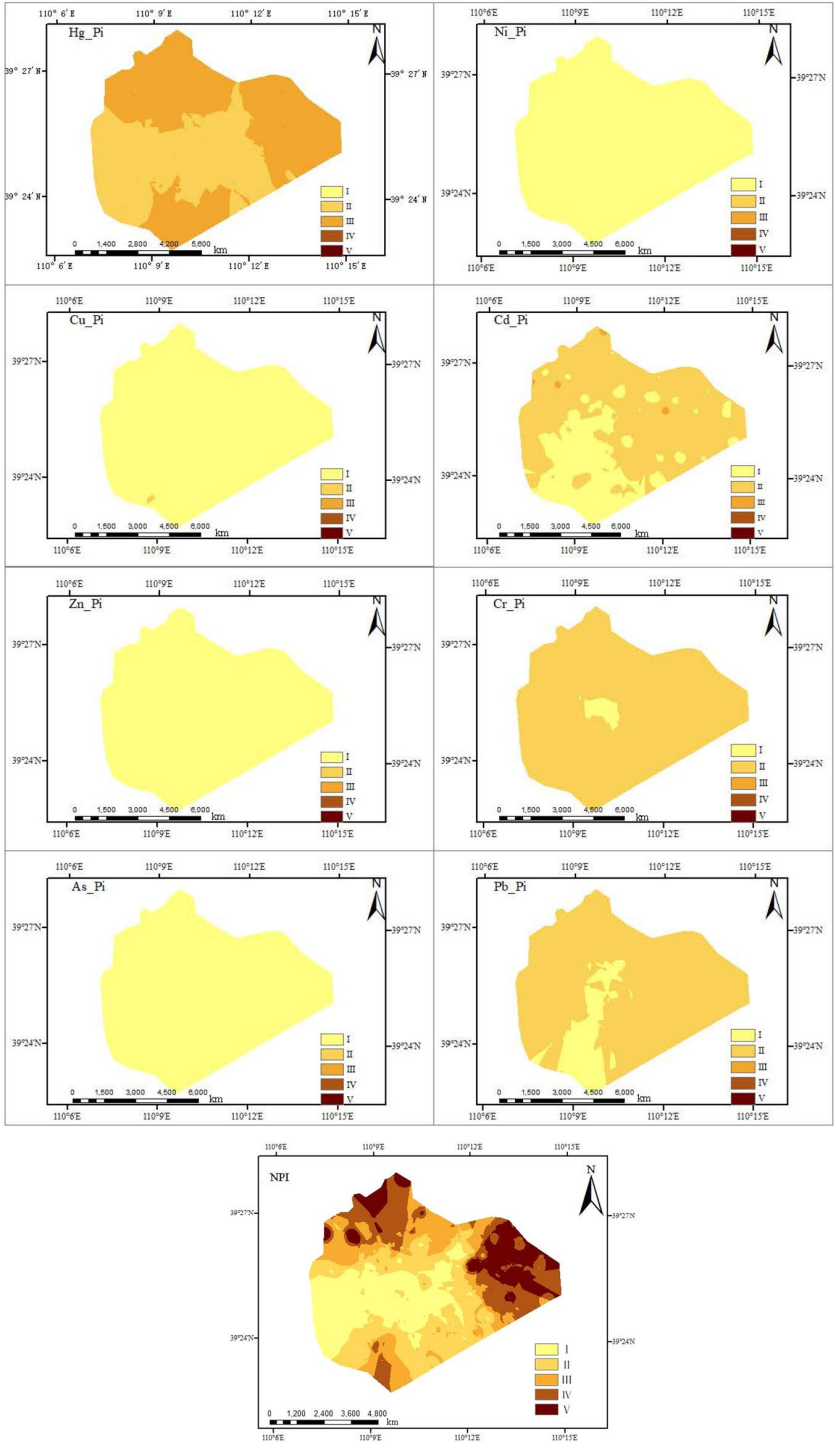
Spatial distribution of *P*_i_ and *NPI* of heavy metal in the study area (created by Arcgis 10.8, http://desktop.arcgis.com/cn/).

The spatial distribution pattern of P_i_ and NPI values of eight heavy metals in the surface soil of Shigetai Coal Mine is drawn by ARCGIS 10.8 software based on GIS technology and geostatistical analysis (Fig. 2).

As can be seen from Fig. 2, the heavy metal Hg in the surface soil within the research area ranks top in the pollution degree and area. The heavy metal Hg is at the moderate pollution level in the east, south and north of the research area, while it is at the mild pollution level in the west and middle of the research area. The heavy metal Cr in the research area ranks second in the pollution area. The element Cr is at the clean level in the middle of the research area, but at the mild pollution level in other areas, with small clean area. The heavy metal Pb in the research area ranks third in the pollution area. The element Pb is at the clean level in the south and middle (with small clean area) of the research area, but at the mild pollution level in other areas, with large area of mild pollution. The heavy metal Cd in the research area ranks fourth in the pollution area. The element Cd is at the clean level in the northwest (with small clean area) and south of the research area, but at the mild pollution level in other areas. Other heavy metal elements in the research area are at the clean level. Relevant researches show that the heavy metal pollution in soil of the coal mine is mainly caused by various mining activities^46^. Compared with natural soils, the elevated concentrations of heavy metals in the mining-affected soils were also reported elsewhere, e.g., Bangladesh^38^and India^47^.

According to the study results of Sun and Li et al.^48^, in the process of coal mining, a large amount of coal gangue and fly ash is produced. During the process of rain leaching, many heavy metals, such as Pb, Hg, Crand Cd are released from coal gangue and fly ash. This is the most important sources of Pb, Hg, Cd, and Cr. In addition, traffic activities such as the wear of motor vehicle brake blocks and other parts, exhaust emissions, etc. are also one of the sources of heavy metal (Hg) pollution in soil^49^. Combined with the actual situation of the research area, the research area is adjacent to the Shaanxi-Inner Mongolia border in the east, so the exhaust emissions of motor vehicles may also be another cause of Hg pollution in the research area. Based on the spatial distribution pattern of NPI of heavy metal in the research area, its distribution status is basically consistent with the spatial distribution of P_i_ of heavy metal Hg.

### Evaluation of potential ecological risks of heavy metal pollution in soil

By taking the soil background value of Shaanxi Province as the reference value, the single potential ecological risk index (*E*) and RI of heavy metals at each sampling point in Shigetai Coal Mine are calculated, and the ecological risk is evaluated according to the classification standard of potential ecological risks. It can be seen from Table 7 that the average value of E of the heavy metal in the surface soil within the research area is Hg(81.01), Cd(34.13), As(5.90), Pb(5.56), Cu(2.83), Ni(2.80), Cr(2.44) and Zn(0.68) in descending order. Except the heavy metal Hg, the average value of potential ecological risk index of other heavy metals in the surface soil within the research area is less than 40, so it is at a mild pollution level. The potential ecological risk of the element Hg ranges from 27.30 to 161.90, with an average value of 81.01, so it is in a relatively high ecological risk. It can be seen that Hg is the most important ecological risk factor in the research area. RI in the research area ranges from 53.44 to 6400.00 and the average value of comprehensive potential ecological risk index is 1336.49, so it is in an extremely high ecological risk.

**Table 7:** Evaluation of ecological risk of heavy metals in the surface soil within the research area.

Parameter	*E*								*RI*
	Hg	Cd	As	Pb	Cr	Zn	Ni	Cu	
Minimum	27.30	17.21	2.40	2.76	1.30	0.27	0.75	1.16	53.44
Maximum	161.90	88.11	16.58	10.00	3.80	1.01	4.88	10.81	6400.00
Average	81.01	34.13	5.90	5.56	2.44	0.68	2.80	2.83	1336.49
Pollution class	High	Low	Low	Low	Low	Low	Low	Low	Extremely high

E stands for the individual potential ecological risk index.

### Spatial distribution pattern of potential ecological risks of heavy metal pollution in soil

**Figure 3:**
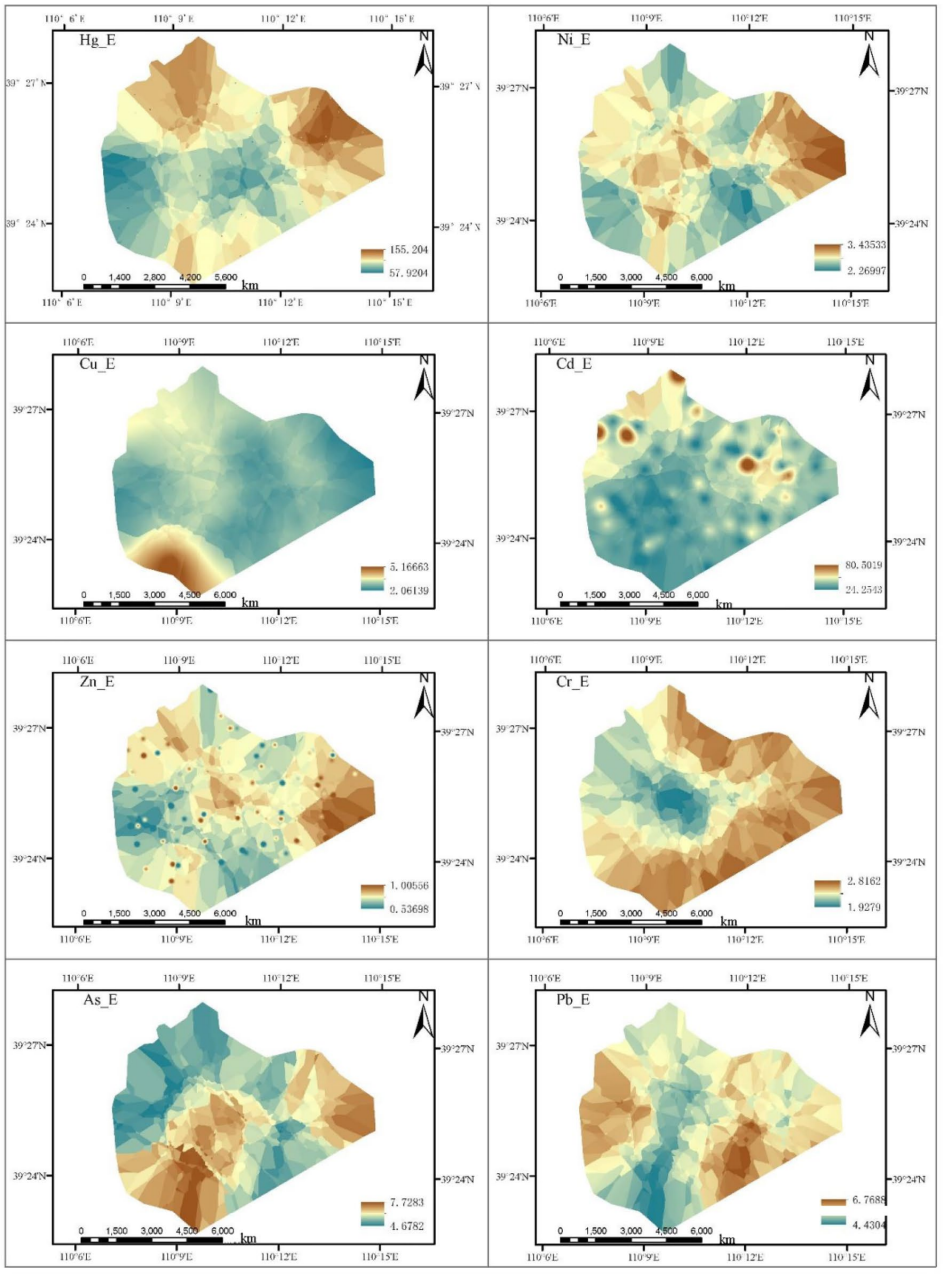
Spatial distribution of single ecological risk index (E) of heavy metals (created by Arcgis 10.8, http://desktop.arcgis.com/cn/).

The spatial distribution pattern of E and RI of heavy metals in the surface soil of Shigetai Coal Mine is as shown in Fig. 3. It can be seen from Fig. 3 that the spatial distribution of E value of the element Hg is basically consistent with the spatial distribution of P_i_, the distribution area of Hg is relatively large, and the potential ecological risk index is in the moderate risk, indicating that Hg is the main ecological risk factor in this mine area. The spatial distribution of E values of other heavy metals Cd, As, Pb, Cr, Zn, Ni and Cu is quite different. Among them, Ni, Zn and Pb are mainly distributed in the east and midwest of the mine area, Cr and As are mainly distributed in the east and southeast of the mine area, the heavy metal Cu is only distributed in the south of the mine area, and the heavy metal Cd is mainly distributed in the northwest and in the east (small area) of the mine area.

**Figure 4:**
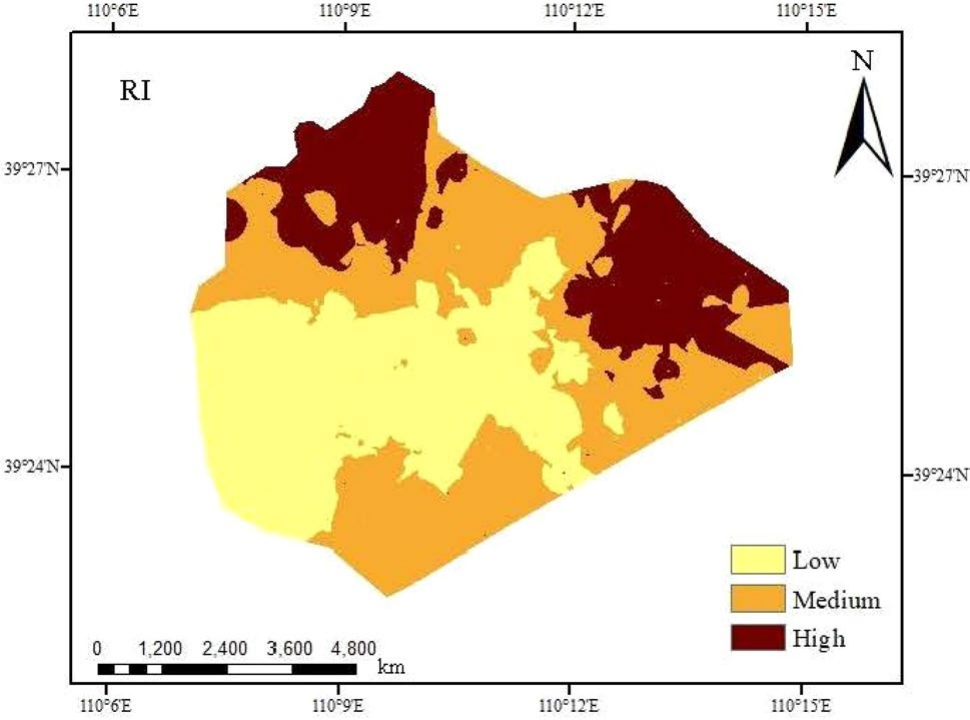
Spatial distribution of RI of heavy matals(created by Arcgis 10.8, http://desktop.arcgis.com/cn/).

As can be seen from Fig. 4, the ecological risk index is high in the east and north of the research area from the spatial distribution pattern of RI in the research area, showing extremely strong risks, while it is in the slight risk in the middle and west, and moderate risk in other areas. On the whole, the spatial distribution pattern of RI values of heavy metals in the research area shows an obvious horizontal zonal distribution pattern, which is basically consistent with the distribution pattern of NPI.

### Ecological risk warning of heavy metal pollution in soil

Based on the ecological risk evaluation, the ecological risk warning evaluation emphasizes the research on possible risk warning in ecosystem. The warnings for the ecological hazards caused by surface soil heavy metal pollution in the Shigetai Coal Mine in northern Shaanxi are evaluated based on the classification standard of ecological risks proposed by Rapant and Kordik^36^. It can be seen from Table 8 that the average values of *I*_ER_ of eight elements including Hg, Cd, As, Pb, Cr, Zn, Ni and Cu are 1.03, 0.14, -0.41, 0.11, 0.22, -0.32, -0.44 and -0.43, respectively. Among them, Hg is in the slight warning status, Cd, Pb and Cr are in the warning status, and As, Zn, Ni and Cu are in no warning status. *I*_ER_ values in the research area range from -34.80 to 81.00 and the average value is -1.13, so it is in no warning status.

**Table 8:** Warning of ecological risks of heavy metals in surface soil within the research area.

Parameter	$I_{{{\text{ERi}}}}$								$I_{{{\text{ER}}}}$
	Hg	Cd	As	Pb	Cr	Zn	Ni	Cu	
Minimum	− 0.32	− 0.43	− 0.76	− 0.45	− 0.35	− 0.73	− 0.85	− 0.77	− 34.80
Maximum	3.05	1.94	0.66	1.00	0.90	0.01	− 0.02	1.16	81.00
Average	1.03	0.14	− 0.41	0.11	0.22	− 0.32	− 0.44	− 0.43	− 1.13
Warning class	Low	Early	No	Early	Early	No	No	No	No

**Figure 5:**
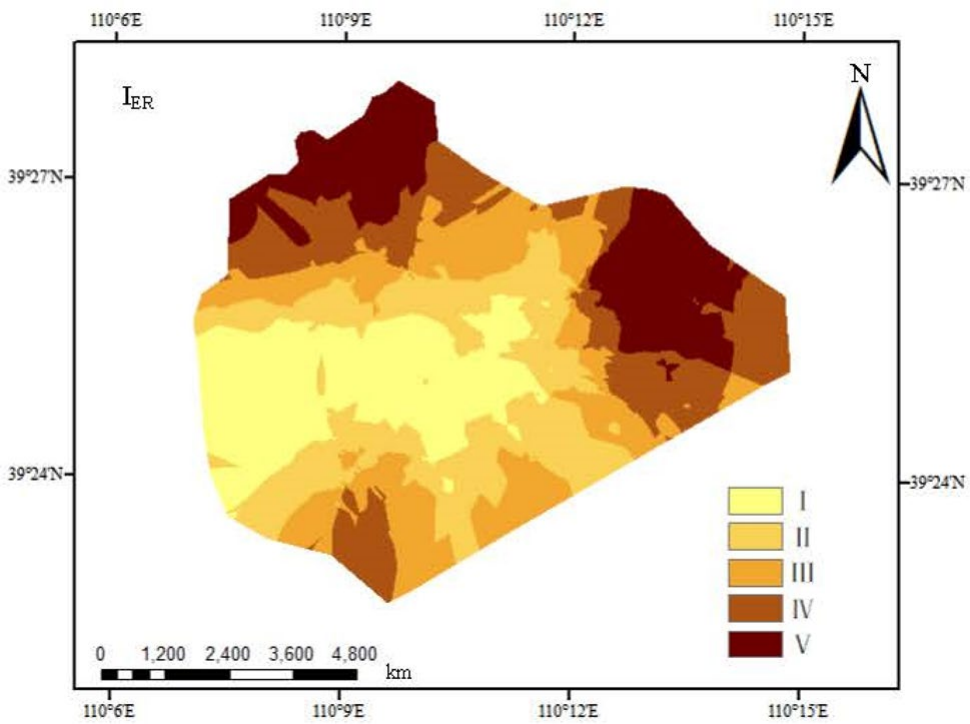
Ecological risk warning assessment of heavy metals in the study area (created by Arcgis 10.8, http://desktop.arcgis.com/cn/).

Based on the spatial distribution of *I*_ER_ values (Fig. 5), the northern and eastern areas in the research area show serious warning, the transition area between the east and the north shows moderate warning to slight warning, the western and central areas show no warning, and other areas show warning to slight warning. The areas with high ecological risk warning values are mainly distributed in the east and north, with relatively serious pollution, showing relatively obvious zonal distribution law.

## Conclusion

1. The contents of Hg, Cd, As, Pb, Cr, Zn, Ni and Cu in the soil within the research area vary from 0.043 to 0.255, 0.44 to 2.23, 2.66 to 18.40, 11.80 to 42.80, 40.50 to 118.60, 18.90 to 70.10, 4.31 to 28.10, 4.96 to 46.25 mg/kg, respectively. Among them, the average contents of heavy metals Hg, Cd, Pb and Cr are 2.03, 1.36, 1.11 and 1.23 times of the soil background values in Shaanxi Province respectively and the average contents of other heavy metals are lower than the soil background values in Shaanxi Province; based on the coefficient of variation, Hg and Cd show moderate variation while As, Pb, Cr, Zn, Ni and Cu show strong variation; the skewness coefficient and kurtosis coefficient of Cd, As and Cu in the soil within the research area are relatively high, and these elements are accumulated in large amounts.
2. The analysis of single factor pollution of heavy metals in the soil within the research area shows that the heavy metal Hg pollutes the soil seriously, so it is at the moderate pollution level, and it is mainly distributed in the east and north of the research area as well as a small area in the south, while three elements including Cd, Pb and Cr are at the mild pollution level. The analysis of Nemerow pollution index method shows that Nemerow pollution index in the research area reaches level III due to mining activities, and Hg is the most important pollution factor.
3. The average value of potential ecological risk index of heavy metals in soil within the research area is Hg(81.01), Cd(34.13), As(5.90), Pb(5.56), Cu(2.83), Ni(2.80), Cr(2.44) and Zn(0.68), respectively. The potential ecological risk of the heavy metal Hg ranges from 27.30 to 161.90, with an average value of 81.01, so it is in a relatively high ecological risk. Other heavy metals are in the low risk;.
4. From the perspective of spatial distribution, the eastern and northern ares in the research area are in the high risk, the central and western areas are in the low risk, and the other areas are in the medium risk; the areas with high ecological risk warning values are mainly distributed in the east and north, with relatively serious pollution, and the whole research area shows relatively obvious zonal distribution law. Due to the long-term human activities, the spatial heterogeneity of heavy metal pollution is obviously enhanced, and the potential risks may be increased beyond our expectations. Therefore, the ecological risks and human health risks of heavy metal pollution in this area shall be comprehensively evaluated in the future study, so as to provide a basis for the prevention and control strategies of heavy metal pollution in coal mine areas.

## Data Availability

The datasets analysed during the current study available from the corresponding author on reasonable request.
